# Indirect Effects of Municipal Public Health Nurse Workforce on Cancer Standardized Mortality Ratios Mediated by Cancer Screening Rates

**DOI:** 10.1111/phn.13451

**Published:** 2024-10-16

**Authors:** Shimpei Kodama, Rika Hinokuma

**Affiliations:** ^1^ Department of Comprehensive Community‐based Nursing Science, School of Health Sciences, Faculty of Medicine Kagoshima University Kagoshima Japan

**Keywords:** cancer screening, ecological study, municipalities, public health nurse, standardized mortality ratio, structural equation modeling, workforce

## Abstract

**Objective:**

This study examined the indirect effects of the number of Japanese municipal public health nurses (PHNs) on cancer standardized mortality ratios (SMRs), using cancer screening and diagnostic follow‐up rates as mediators.

**Design:**

Ecological study using municipalities as the unit of analysis

**Measurements:**

Aggregate, municipal‐level government data were analyzed using a linear model with empirical Bayes estimates of SMRs (EBSMRs) for gastric, colorectal, and lung cancers as the dependent variables, and the number of PHNs, cancer screening rate, diagnostic follow‐up rate, and adjustment variables as independent variables. Structural equation modeling (SEM) was used to examine the indirect effects of PHNs.

**Results:**

Cancer screening rates were significantly negatively associated with EBSMR, except for gastric cancer in women. No significant association was observed between the EBSMR and diagnostic follow‐up rates. SEM revealed a significant indirect effect of the number of PHNs, most of which was due to the cancer screening rate.

**Conclusions:**

From a population‐based public health perspective, increasing the number of PHNs and focusing on improving cancer screening rates may effectively reduce cancer SMRs.

## 1 Introduction

Cancer is the leading cause of death worldwide (World Health Organization 2023), and cancer prevention is increasingly important. In Japan, cancer and malignant neoplasms have been the leading causes of death since 1981 (Ministry of Health, Labour, and Welfare 2019), making cancer control an important issue. Cancer control requires not only improved cancer treatment methods, but also the promotion of cancer prevention, early detection, and early treatment to improve patients’ quality of life and reduce the burden of rising medical costs. In Japan, public health nurses (PHNs) are among the professionals who contribute to cancer control through activities aimed at improving the health status of entire communities.

PHNs in Japan have national qualifications distinct from registered nurses (RNs), and many PHNs are employed by prefectures and municipalities as civil servants (approximately 70% of the 60,000 employed PHNs in Japan are civil servants) (Ministry of Health, Labour, and Welfare 2022). Japanese PHNs have provided various health services over time, ranging from direct services such as maternal, child, and adult health screening and tuberculosis control, to community‐wide services such as the assessment of comprehensive health problems, establishment of healthcare systems, and recommendations for policies in aging communities (Iwasaki‐Motegi and Naruse 2020; Kanbara et al. 2017; Murashima et al. 1999). Recently, many services have been provided by municipalities rather than by prefectures, and PHNs in municipal health promotion departments have played an increasingly important role. However, few studies have examined the relationship between the PHN workforce and health indicators (Kobayashi et al. 2015; Kondo et al. 2005; Mishina, Hilton, and Takayama 2013; Nomura et al. 2017). Therefore, using Donabedian's structure‐process‐outcome framework to assess the quality of healthcare services (Donabedian 1980), the authors have examined the effect of the PHN workforce in each municipality in Japan on the standardized mortality ratio (SMR), especially the cancer SMR, from both cross‐sectional and longitudinal perspectives (Kodama et al. 2019; Kodama, Uwatoko, and Koriyama 2023). The results of these previous studies suggested that activities conducted by an increased number of PHNs may reduce cancer mortality by promoting cancer screening among residents (Kodama et al. 2023). Therefore, the number of PHNs (the structure component of the Donabedian's framework) was hypothesized to have an indirect effect on cancer SMR (the outcome component of the Donabedian's framework) through the cancer screening rate (the process component of the Donabedian's framework) as a mediator. In addition, diagnostic follow‐up is recommended for individuals with positive cancer screening results, with subsequent treatment initiation for positive follow‐up results. The activities of PHNs include recommendations for screening and diagnostic follow‐ups, both of which may mediate the relationship between the number of PHNs and cancer SMRs. Therefore, both cancer screening and diagnostic follow‐up rates are considered mediators. However, while many studies have shown a relationship between cancer screening rates and cancer mortality (Lee et al. 2006, 2007; Miyamoto et al. 2007; Nishii et al. 2001; Sagawa et al. 2001; Tanaka et al. 2023; Tsukada et al. 2001), few quantitative studies have reported on the relationship between the number of PHNs and cancer screening rates (Hatano et al. 2013; Takaku 2011), and none have examined the indirect effect of cancer screening or diagnostic follow‐up rates. A more detailed examination of the relationship between the PHN workforce and cancer‐related deaths is important for making appropriate policy decisions regarding PHN training and deployment.

Therefore, this study examined the indirect effects of the number of Japanese municipal PHNs on cancer SMRs, using cancer screening and diagnostic follow‐up rates as mediators.

## 2 Methods

### 2.1 Study Design

Ecological study using municipalities as the unit of analysis.

### 2.2 Data Sources

Data were collected from the government statistics portal e‐Stat (Statistics Bureau 2008), including the Population Census; Specific Report of Vital Statistics; Report on Regional Public Health Services and Health Promotion Services; Survey of Physicians, Dentists, and Pharmacists; Dynamic Survey of Medical Institutions; and Survey of Institutions and Establishments for Long‐term Care. Census data were obtained from the Statistics Bureau of the Ministry of Internal Affairs and Communications. Other data were collected from the Ministry of Health, Labor, and Welfare (MHLW).

### 2.3 Study Sample

The study sample consisted of Japanese municipalities listed in the latest Specific Report of Vital Statistics (FY2013–FY2017). Of the 1741 municipalities, eight in which evacuation orders had not been lifted in some areas because of the nuclear power plant accident caused by the Great East Japan Earthquake were excluded. The number of participants analyzed differed according to the cancer type because of missing cancer screening data.

### 2.4 Outcomes

The outcomes were SMRs for gastric, colorectal, and lung cancers (C16, C18–20, and C33–34 of the International Classification of Diseases, 10th revision [ICD‐10]), respectively). These three cancers are among the five cancers (gastric, colorectal, lung, breast, and cervical cancers) for which the Japanese government promotes screening under the Health Promotion Law and for which mortality statistics are available.

SMRs are difficult to interpret in small municipalities where the expected number of deaths is small and the effect of random variation in the number of deaths is large (Elliott et al. 2001). To address this issue, we used the empirical Bayes estimate of SMR (EBSMR), which is a Bayesian method used to smooth SMR values based on the spatial similarity of the surrounding areas (Lawson 2018). The present study used the 2015 EBSMR for each municipality published in the Specific Report of Vital Statistics in FY2013–FY2017. These EBSMRs were calculated using the 2015 population and the 2013–2017 mortality data. Although the 2020 EBSMR was published in the latest Specific Report of Vital Statistics in FY2018–FY2022, only the EBSMR for colorectal cancer was used for sensitivity analysis in the present study because the method and target age for gastric cancer screening were changed in 2016, which affected the EBSMR for gastric cancer. Moreover, the prevalence of coronavirus disease 2019 (COVID‐19) in Japan in 2020 is expected to affect the EBSMR for lung cancer (Khoury et al. 2022).

### 2.5 Influencing Factor, Mediator, and Adjustment Factor

The primary influencing factor was the number of PHNs per 100,000 population, including both full‐time and part‐time PHNs, based on a previous study (Kodama et al. 2023). The mediators were the cancer screening and diagnostic follow‐up rates. The cancer screening rate was defined as the proportion of individuals who were screened among those aged ≥ 40 years for whom screening was recommended by the Japanese government. The diagnostic follow‐up rate was defined as the proportion of individuals who received a diagnostic follow‐up in the following year among those with positive cancer screening results.

Our previous study did not consider the time lag between the PHN workforce and mortality (Kodama et al. 2023). Therefore, in the present study, the number of PHNs per population and cancer screening and diagnostic follow‐up rates were calculated based on the combined data from 2011 and 2012 (before 2013–2017, which were used to calculate EBSMRs) to account for the time lag. We did not use data from 2010 or earlier because much data were missing in this year owing to the Great East Japan Earthquake, and the difficulty in matching data from 2010 or earlier owing to the large number of municipal mergers that occurred before 2010.

As described previously (Kodama et al. 2019, 2023), health and welfare resources, the structural components of Donabedian's framework, were used as adjustment factors affecting the relationship between the number of PHNs, cancer screening rates, and outcomes.  The number of health and welfare resources was defined as the number of physicians, medical clinics, and general hospitals in a secondary healthcare area to which the municipality belongs, and welfare facilities for the elderly requiring long‐term care (all per 100,000 people in 2015). We added a dummy variable indicating whether the municipality was a “health center designated city” as an adjustment factor for the municipality type as these cities are exceptionally responsible for prefectural health services due to their large size. Moreover, their PHN assignments and working styles differ from those of general municipalities because the roles of municipal and prefectural PHNs are different (Iwasaki‐Motegi and Naruse 2020).

### 2.6 Analysis Strategy

After examining the descriptive statistics and bivariate associations, we examined the relationships between cancer EBSMR and the number of PHNs and cancer screening and diagnostic follow‐up rates using a linear model. The cancer EBSMR was used as the dependent variable, while the number of PHNs per 100,000 population, cancer screening rate, diagnostic follow‐up rate, adjustment variables (the number of physicians, medical clinics, general hospitals in the secondary healthcare area to which the municipality belonged, welfare facilities for older adults requiring long‐term care, all per 100,000 population, and municipality type), and population were used as the independent variables. After fitting the population distribution by municipality in Japan to a log‐normal distribution model (Inoue 1998), municipalities with small populations had more PHNs per population than those with large populations (Hayakawa 2006). Therefore, a logarithmic transformation was performed on the natural logarithm for the population and number of PHNs per 100,000 population. Additionally, because the status of deaths in a region varies by prefecture (Ministry of Health, Labour, and Welfare 2017a), variation to the linear model was added as a random effect for 47 prefectures. The Akaike information criterion (AIC) was used to assess the appropriateness of including cancer screening and diagnostic follow‐up rates in the models. A mixed linear model from IBM SPSS Statistics for Windows, version 28.0, was used to examine the linear model.

Structural equation modeling (SEM) was then used to examine the indirect effect of the number of PHNs on cancer EBSMR, mediated by cancer screening and diagnostic follow‐up rates. In this SEM, the number of PHNs and adjustment variables were all assumed to be correlated, and the differences among the 47 prefectures were entered as 46 dummy variables that were independent of each other. As a sensitivity analysis of the indirect effects using SEM, a similar analysis was performed for colorectal cancer, a cancer for which conditions are the same and comparable, using data collected 5 years later; that is, the EBSMR and adjusted variables in 2020 and the number of PHNs, cancer screening rates, and diagnostic follow‐up rates in 2016 and 2017. IBM SPSS Amos 21 was used to perform the SEM, and bootstrap resampling (2000 bootstrap samples) was used to examine the statistical significance of the total indirect effect using the cancer screening and diagnosis follow‐up rates as mediating variables. Each analysis was conducted according to participant sex. The significance level was set at *p* < 0.05.

## 3 Results

### 3.1 Descriptive Statistics and Bivariate Associations

The descriptive statistics are presented in Table 1. The EBSMR for lung cancer tended to be lower in women than in men, with no other differences between sexes. A closer look at the EBSMR for lung cancer in women showed that it was higher in health center‐designated cities than in general municipalities (106.0 and 98.8, respectively), a trend not observed in other EBSMRs. The screening rate for lung cancer was the highest, followed by those for colorectal and gastric cancers. The diagnostic follow‐up rate tended to be high for gastric and lung cancers, and low for colorectal cancer.

**TABLE 1:** Descriptive statistics (*n* = 1730).

	Men		Women	
	Mean (SD) or *n* (%)			
EBSMRs (2015)				
Gastric cancer	99.2	(16.9)	99.2	(17.1)
Colorectal cancer	99.2	(14.0)	98.9	(12.5)
Lung cancer	100.3	(12.6)	96.9	(14.9)
Cancer screening rate (2011 and 2012)				
Gastric cancer	0.15	(0.10)	0.14	(0.09)
Colorectal cancer	0.22	(0.12)	0.22	(0.11)
Lung cancer	0.25	(0.16)	0.26	(0.17)
Diagnostic follow‐up rate (2011 and 2012)				
Gastric cancer	0.80	(0.14)	0.86	(0.13)
Colorectal cancer	0.69	(0.15)	0.74	(0.15)
Lung cancer	0.80	(0.17)	0.84	(0.16)
Number of PHNs (2011 and 2012)^a^		41.8	(39.8)	
Numbers of health care resources (2015)^a^				
Physicians		162.6	(176.3)	
Medical clinics		78.1	(53.5)	
General hospitals in a secondary healthcare area		7.2	(3.5)	
Welfare facilities for the elderly requiring long‐term care		11.3	(12.2)	
Population (2015)		73,399.4	(188,529.8)	
Type of municipality (2015): health center‐designated cities		92	(5.3%)	

Abbreviations: EBSMR, empirical Bayes estimate of standardized mortality ratio; PHN, public health nurse.

^a^ Per 100,000 population.

Table 2 shows the results of the correlation analysis between the EBSMR and the number of PHNs, cancer screening rate, and diagnostic follow‐up rate for each cancer. The EBSMR was significantly negatively correlated with gastric cancer in both men and women, in which the higher the number of PHNs, the lower the EBSMR. A similar although non‐significant trend was observed for colorectal cancer in both men and women, with an inverse significant positive correlation for lung cancer in men. This positive correlation was attributed to confounding by prefectural differences as it was no longer significant after adjusting for these differences. Higher screening rates were significantly negatively correlated with lower EBSMRs for colorectal cancer in men and lung cancer in both men and women. Higher screening rates were positively correlated with higher EBSMRs for gastric cancer in both men and women. The positive correlation between the EBSMR and screening rate was also attributed to confounding by prefectural differences, as the sign of the coefficient was reversed after adjusting for prefectural differences. Finally, a higher diagnostic follow‐up rate was positively correlated with a higher EBSMR for gastric cancer in both men and women and lung cancer in men.

**TABLE 2:** Bivariate correlation for cancer EBSMR.

	EBSMR in men			EBSMR in women		
	(*n*)	*R*	*p* value	(*n*)	*R*	*p* value
Gastric cancer						
Number of PHNs^a^	(1725)	−0.122	< 0.001	(1725)	−0.127	< 0.001
Cancer screening rate	(1725)	0.117	< 0.001	(1725)	0.097	< 0.001
Diagnostic follow‐up rate	(1716)	0.068	0.005	(1716)	0.095	< 0.001
Colorectal cancer						
Number of PHNs^a^	(1730)	−0.042	0.080	(1730)	−0.047	0.050
Cancer screening rate	(1730)	−0.115	< 0.001	(1730)	−0.034	0.153
Diagnostic follow‐up rate	(1726)	−0.043	0.072	(1725)	0.020	0.406
Lung cancer						
Number of PHNs^a^	(1667)	0.113	< 0.001	(1667)	−0.033	0.181
Cancer screening rate	(1667)	−0.093	< 0.001	(1667)	−0.285	< 0.001
Diagnostic follow‐up rate	(1636)	0.084	< 0.001	(1629)	−0.017	0.481

Abbreviations: EBSMR, empirical Bayes estimate of standardized mortality ratio; PHN, public health nurse; *R*, Pearson's product‐moment correlation coefficient.

^a^ Per 100,000 population (log‐transformed).

The number of PHNs and cancer screening rates showed significant positive correlations for both men and women for all cancer types, with a higher number of PHNs resulting in higher screening rates (correlation coefficients: 0.227–0.356). The positive correlation between the number of PHNs and diagnostic follow‐up rates indicated that the higher the number of PHNs, the higher the diagnostic follow‐up rate (correlation coefficients: 0.073–0.172), except for gastric cancer in men.

### 3.2 Linear Model and SEM

Table 3 shows the results of the linear model analysis examining the association between EBSMR and the number of PHNs per 100,000 people and cancer screening and diagnostic follow‐up rates for each cancer type. High cancer screening rates were significantly negatively associated with lower EBSMR for all cancers except gastric cancer in women. Diagnostic follow‐up rates showed no significant associations. In all linear models, the AIC was lower than that in the models excluding cancer screening and diagnostic follow‐up rates, indicating the appropriateness of including these rates in the models.

**TABLE 3:** Results of multivariate linear model analysis with cancer EBSMR as the dependent variable.

	Men		Women	
	Coefficient^b^	*p* value	Coefficient^b^	*p* value
Gastric cancer	(*n* = 1716)		(*n* = 1716)	
Number of PHNs^a^	−1.60	0.040	−2.07	0.010
Cancer screening rate	−6.78	0.015	−5.91	0.067
Diagnostic follow‐up rate	1.05	0.575	0.16	0.937
	(AIC = 12,768.3)		(AIC = 12,852.2)	
Colorectal cancer	(*n* = 1726)		(*n* = 1725)	
Number of PHNs^a^	−1.13	0.096	−0.44	0.496
Cancer screening rate	−13.38	< 0.001	−12.01	< 0.001
Diagnostic follow‐up rate	1.97	0.206	1.01	0.510
	(AIC = 12,458.5)		(AIC = 12,294.6)	
Lung cancer	(*n* = 1636)		(*n* = 1629)	
Number of PHNs^a^	−0.45	0.491	−0.90	0.234
Cancer screening rate	−4.24	0.005	−7.68	< 0.001
Diagnostic follow‐up rate	0.52	0.664	−0.68	0.647
	(AIC = 11,498.6)		(AIC = 11,892.4)	

Abbreviations: AIC, Akaike information criterion; EBSMR, empirical Bayes estimate of standardized mortality ratio; PHN, public health nurse.

^a^ Per 100,000 population (log‐transformed).

^b^ Coefficients are adjusted for random effects by prefecture and fixed effects by the number of physicians, medical clinics, general hospitals in the secondary healthcare area to which the municipality belongs, welfare facilities for older adults requiring long‐term care (all per 100,000 population), type of municipality, and population (log‐transformed).

**FIGURE 1:**
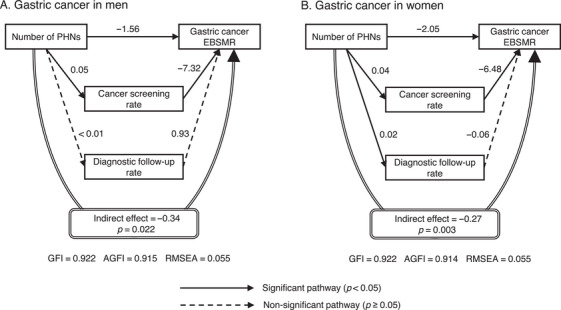
Main path coefficients and indirect effects for gastric cancer according to structural equation modeling (SEM). The number of PHNs per 100,000 people was log‐transformed. The coefficients were adjusted for the number of physicians, medical clinics, general hospitals in the secondary healthcare area to which the municipality belonged, welfare facilities for older adults requiring long‐term care (all per 100,000 people), municipality type, population (log‐transformed), and prefecture dummy variables. Except for the prefecture dummy variables, the number of PHNs and adjustment variables were assumed to be correlated. Abbreviations: AGFI, adjusted goodness‐of‐fit index; EBSMR, empirical Bayes estimate of standardized mortality ratio; GFI, goodness‐of‐fit index; PHN, public health nurse; RMSEA, root mean square error of approximation.

**FIGURE 2:**
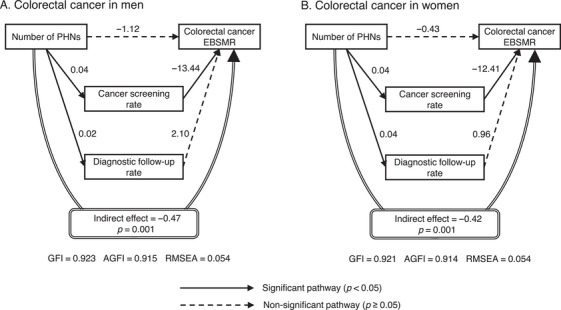
Main path coefficients and indirect effects for colorectal cancer according to structural equation modeling (SEM). The number of PHNs per 100,000 people was log‐transformed. The coefficients were adjusted for the number of physicians, medical clinics, general hospitals in the secondary healthcare area to which the municipality belonged, welfare facilities for older adults requiring long‐term care (all per 100,000 people), municipality type, population (log‐transformed), and prefecture dummy variables. Except for the prefecture dummy variables, the number of PHNs and adjustment variables were assumed to be correlated. Abbreviations: AGFI, adjusted goodness‐of‐fit index; EBSMR, empirical Bayes estimate of standardized mortality ratio; GFI, goodness‐of‐fit index; PHN, public health nurse; RMSEA, root mean square error of approximation.

**FIGURE 3:**
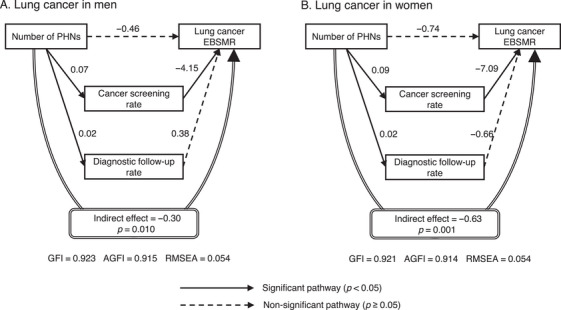
Main path coefficients and indirect effect for lung cancer according to structural equation modeling (SEM). The number of PHNs per 100,000 people was log‐transformed. The coefficients were adjusted for the number of physicians, medical clinics, general hospitals in the secondary healthcare area to which the municipality belonged, welfare facilities for older adults requiring long‐term care (all per 100,000 people), municipality type, population (log‐transformed), and prefecture dummy variables. Except for the prefecture dummy variables, the number of PHNs and adjustment variables were assumed to be correlated. Abbreviations: AGFI, adjusted goodness‐of‐fit index; EBSMR, empirical Bayes estimate of standardized mortality ratio; GFI, goodness‐of‐fit index; PHN, public health nurse; RMSEA, root mean square error of approximation.

The main path coefficients and indirect effects from the SEM analysis are shown in Figures 1, 2, 3. Significant associations were observed between a higher number of PHNs and a higher cancer screening rate and between a higher cancer screening rate and a lower EBSMR for all cancers in men and women. The follow‐up rate was also positively associated with the number of PHNs for all cancers, except for gastric cancer in men. However, the follow‐up rate was not significantly associated with the EBSMR. All cancers showed a significant indirect effect mediated by the cancer screening and diagnostic follow‐up rates, in which the lower the EBSMR, the higher the number of PHNs. For all indirect effects, the effect was due to the pathway mediated by the cancer screening rate. The direct effect of the number of PHNs on the EBSMR, excluding the indirect effects, was significantly associated with gastric cancer in men and women.

**FIGURE 4:**
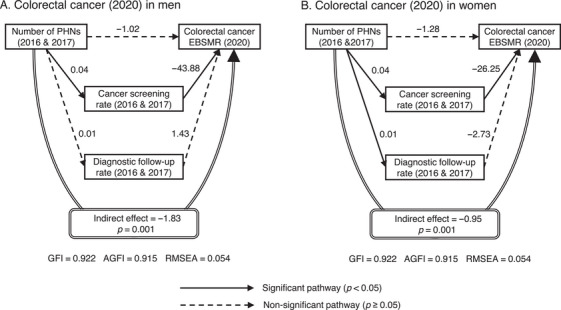
Sensitivity analysis according to structural equation modeling (SEM) for colorectal cancer EBSMR based on data from 5 years later. The number of PHNs per 100,000 people was log‐transformed. The coefficients were adjusted for the number of physicians, medical clinics, general hospitals in the secondary healthcare area to which the municipality belonged, welfare facilities for older adults requiring long‐term care (all per 100,000 people), municipality type, population (log‐transformed), and prefecture dummy variables. Except for the prefecture dummy variables, the number of PHNs and adjustment variables were assumed to be correlated. Abbreviations: AGFI, adjusted goodness‐of‐fit index; EBSMR, empirical Bayes estimate of standardized mortality ratio; GFI, goodness‐of‐fit index; PHN, public health nurse; RMSEA, root mean square error of approximation.

The results of the sensitivity analysis by SEM using the EBSMR for colorectal cancer in 2020 and the number of PHNs, cancer screening rates, and diagnostic follow‐up rates using the 2016 and 2017 data are shown in Figure 4. Although the relationship between PHNs and diagnostic follow‐up rates in men was not significant, all other associations and indirect effects were similar to those of the 2015 EBSMR for colorectal cancer, indicating the robustness of the study findings.

## 4 Discussion

### 4.1 Municipality Characteristics

The EBSMR for all municipalities was close to 100 for most cancers but was lower for lung cancer in women. Moreover, the EBSMR for lung cancer in women according to municipality type was lower in general municipalities and higher in health center‐designated cities. The effect of smoking on lung cancer is well known, and the smoking rate among women in Japan tends to be higher in urban areas (Tomioka 2020). In an ecological analysis at the municipal level, such as in this study, the effect of the individual level of a large city, such as a health center‐designated city, is reduced. Therefore, the effect of high smoking rates on lung cancer risk among women in urban areas may be smaller and appear as a lower EBSMR in all municipalities.

The overall cancer screening rates in this study were low, ranging from 14% to 16%. These are below the current target rates of 40% for gastric, lung, and colorectal cancers set by the MHLW (Ministry of Health, Labour, and Welfare 2012). This may be due to the fact that the cancer screening data in this study only included cancer screening provided by municipalities and do not reflect screening provided at workplaces. However, since the activities of municipal PHNs, which were the focus of this study, would naturally be expected to have a strong influence on cancer screening provided by municipalities, we believe that the validity of the results regarding the relationship between municipal PHNs and cancer screening can be guaranteed to some extent. The diagnostic follow‐up rates in this study ranged from 69% to 86%, and although they did not reach the MHLW target of 90% (Ministry of Health, Labour, and Welfare 2017b), they were less discordant than the cancer screening rates.

Cancer screening rates differ according to the cancer type. In Japan, gastric cancer screening is performed using gastrointestinal radiography or endoscopy, lung cancer screening using chest radiography, and colorectal cancer screening using fecal occult blood tests. Chest x‐rays are less invasive and can be performed immediately at mass screening sites, whereas the fecal occult blood test is less invasive but relatively time‐consuming, requiring advanced preparation, such as the collection of stool samples. Gastrointestinal radiography and endoscopy are relatively invasive. Gastrointestinal endoscopy, in particular, is available in a limited number of facilities and often requires individual examinations, making it difficult to obtain. These differences in screening burdens may have contributed to the differences in screening rates.

### 4.2 Indirect Effects of the PHN Workforce

The results of the SEM analysis showed that an increase in the municipal PHN workforce in Japan had an indirect effect on the decrease in cancer SMRs through increases in various cancer screening rates. In contrast, except for male patients with gastric cancer, while an increase in the PHN workforce resulted in an increased diagnostic follow‐up rate, the diagnostic follow‐up rate did not affect the SMR. These results suggested that from a population‐based public health perspective, increasing the number of PHNs and focusing on improving cancer screening rates may be effective in reducing cancer SMRs. The lack of confirmation of the effect of the diagnostic follow‐up rate on SMRs may be attributed to the relatively high rates in most municipalities. Areas with low diagnostic follow‐up rates may demonstrate the same effect as that of the cancer screening rate. Further studies are needed to determine this effect.

### 4.3 Direct Effects of the PHN Workforce on Gastric Cancer SMR

The linear model analysis showed a relationship between lower SMR and a higher number of PHNs among men and women with gastric cancer. The results of the SEM analysis also showed a significant relationship as a direct effect. These results suggested that the relationship between the PHN workforce and death from gastric cancer differs from the indirect effect mediated by cancer screening and that an effect of PHN prevention activities may be present, which differs from those of lung and colorectal cancer. For example, previous studies have shown an association between gastric cancer and vegetable and salt intake among Japanese (Kobayashi et al. 2002; Tsugane et al. 2004), whereas meat consumption, which is a risk factor for colorectal cancer, is relatively low in Japan (Takachi et al. 2011). Thus, in Japan, dietary lifestyle counseling by PHNs may be more effective in reducing the risk of gastric cancer than colorectal cancer, and such activities may be a direct effect of PHNs; however, more detailed studies are needed.

### 4.4 Study Limitations

The analysis in this study included only healthcare resources as adjustment factors, consistent with previous studies. Therefore, the effects of factors other than healthcare resources such as actual PHN activities and socioeconomic indicators were not considered. In addition, to account for the time lag between the changes in PHN workforce size and mortality, this study used pre‐outcome data on the number of PHNs and screening rates but was only able to examine the short‐term effects of a time lag of up to 5 years and not longer‐term effects. This study also used SMR as an outcome, which may not reflect the direct effects of PHN activity. Understanding the effects of the PHN workforce in more detail will require an examination of its effects on morbidity, quality of life, and so forth in future studies. However, despite these limitations, the results showed that the increase in the municipal PHN workforce in Japan had an indirect effect on the decrease in cancer SMRs several years later through increases in various cancer screening rates, which may help inform future‐decision making regarding PHN recruitment and appropriate allocation.

## 5 Conclusion

The results of this study demonstrated that an increase in the number of municipal PHNs in Japan had an indirect effect on the decrease in cancer SMRs through increases in various cancer screening rates. While an increase in the number of PHNs also increased the diagnostic follow‐up rate for most cancers, the diagnostic follow‐up rate did not affect the SMR. From a population‐based public health perspective, increasing the number of PHNs and focusing on improving cancer screening rates may effectively reduce cancer SMRs. The direct effect on gastric cancer suggests the effect of prevention activities by PHNs, which differs from the indirect effect mediated by cancer screening.

## Ethics Statement

As this study used only anonymous, processed, and publicly available government statistics, ethical approval by the ethics committee was not required.

## Conflicts of Interest

The authors declare no conflicts of interest.

## Data Availability

The data used in this study are publicly available from the Portal Site of Official Statistics of Japan (e‐Stat) at https://www.e‐stat.go.jp/.
